# Ag-Decorated Vertically Aligned ZnO Nanorods for Non-Enzymatic Glucose Sensor Applications

**DOI:** 10.3390/nano13040754

**Published:** 2023-02-17

**Authors:** Yu-Hsuan Lin, Chandrasekar Sivakumar, Babu Balraj, Gowtham Murugesan, Senthil Kumar Nagarajan, Mon-Shu Ho

**Affiliations:** 1 Institute of Nanoscience, National Chung Hsing University, Taichung City 40227, Taiwan; 2 Department of Physics, National Chung Hsing University, Taichung City 40227, Taiwan; 3 Innovation and Development Center of Sustainable Agriculture (IDCSA), National Chung Hsing University, Taichung 40227, Taiwan; 4 Department of Physics, KPR Institute of Engineering and Technology, Coimbatore 641407, Tamilnadu, India; 5 Postgraduate and Research Department of Physics, Nanotechnology Lab, Kongunadu Arts and Science College, Coimbatore 641029, Tamilnadu, India

**Keywords:** zinc nanorod, silver nanoparticle, hydrothermal method, glucose, biosensor

## Abstract

The non-enzymatic glucose sensing response of pure and Ag-decorated vertically aligned ZnO nanorods grown on Si substrates was investigated. The simple low-temperature hydrothermal method was employed to synthesize the ZnO NRs on the Si substrates, and then Ag decoration was achieved by sputtering. The crystal structure and surface morphologies were characterized by X-ray diffraction, field-emission scanning electron microscopy (FESEM), and transmission electron microscopy (TEM). The Ag incorporation on the ZnO NR surfaces was confirmed using EDS mapping and spectra. Furthermore, the chemical states, the variation in oxygen vacancies, and the surface modifications of Ag@ZnO were investigated by XPS analysis. Both the glucose/ZnO/Si and glucose/Ag@ZnO/Si device structures were investigated for their non-enzymatic glucose sensing performances with different glucose concentrations. Based on EIS measurements and amperometric analysis, the Ag@ZnO-NR-based glucose sensor device exhibited a better sensing ability with excellent stability over time than pure ZnO NRs. The Ag@ZnO NR glucose sensor device recorded 2792 µA/(mM·cm^2^) sensitivity with a lowest detection limit of 1.29 µM.

## 1. Introduction

An abnormally high level of blood glucose, which is more often known as diabetes, is one of the leading causes of death all over the world. The monitoring of glucose levels in the blood is required in order to detect and avert the onset of this potentially fatal condition. The enzyme-based electrochemical detection of glucose has a number of benefits, but it also has a number of limitations, such as a difficult enzyme purification procedure, a high manufacturing cost, a short life period, and mediocre sensitivity as a result of in-direct electron transfer [1,2]. As a result, there is a demand for an electrochemical sensor that does not rely on enzymes, which has led to the development of new materials and methods. In recent years, a significant amount of attention has been paid to the detection of glucose due to the wide range of potential applications in a variety of sectors, including medical and environmental monitoring [3,4]. The oxidation of glucose into gluconolactone is still often accomplished with glucose sensors by the use of an enzymatic reaction that is catalyzed by glucose oxidase (GO_x_) [5]. Recently developed GO_x_-based enzyme sensors have shown particularly impressive levels of sensitivity and selectivity in the detection of glucose levels [6]. Employing metal oxide nanostructures in the non-enzyme-based glucose detector has gained interest due to their high sensitivity and ease of manufacture and integration in a detecting device [2,7]. The usage of nanoparticles (NPs) comes with a plethora of benefits, such as an increase in surface area, a tailored cargo release profile, the regulation of drug pharmacokinetics, decreased toxicity, and a better biological response [8,9]. Ag NPs have the potential to provoke a response from the immune system, leading to the death of inflammatory cells in the host. The functionality and sensitivity of the glucose sensor based on nanostructures are largely reliant on the composition and nanostructure, both of which are essential for the performance. A ZnO nanostructure is one of the attractive possibilities for the non-enzyme-based glucose sensors. This is due to the fact that the ZnO nanostructure is nontoxic and has a low cost of production. Due to its high piezoelectric and pyroelectric capabilities, the lack of a center of symmetry in the wurtzite ZnO crystal makes it a suitable material for mechanical actuators and piezoelectric sensors [10,11,12,13,14,15,16,17]. ZnO has been utilized extensively as a wide-band-gap semiconductor. It is also a versatile material with a wide range of different shapes as well as morphologies, such as nanosprings, nanocombs, nanobelts, nanocages, and nanowires [18,19,20,21,22]. Compared to other noble metals (Au/Pt), silver (Ag) nanoparticles exhibit unique characteristics, including biocompatibility, excellent catalytic activity, low toxicity, antibacterial qualities, and affordability, and, hence, they were chosen in our study to decorate ZnO NRs [23,24,25]. The objective of this work was to evaluate the non-enzymatic glucose detection performance of pure and Ag-decorated ZnO NRs synthesized via a cost-effective synthesis route. The proposed ZnO NRs were synthesized using a low-temperature hydrothermal method and the Ag decoration was achieved by the simple sputtering process. The morphological, structural, and elemental composition, binding features, and vibrational characteristics were evaluated to standardize the synthesized samples via appropriate characterization instruments. The EIS measurements and amperometric study were performed in order to investigate the non-enzymatic glucose sensing performance of the fabricated devices with variable glucose concentrations.

## 2. Materials and Methods

### 2.1. Preparation of the ZnO Seed Layer

An amount of 0.317 g (0.02 M) of zinc acetate dihydrate in 30 mL of methanol was taken and heated to 60 °C while being evenly stirred for 20 min. The temperature of the solution was kept constant at 60 °C, and it was stirred consistently for a period of two hours after the addition of a stabilizer that consisted of 1 mL of ethanolamine. After this period was finished, the solution was left out at room temperature for twenty-four hours so that the zinc oxide seed layer solution could be obtained. Then, using a spin coater, 40 L of zinc oxide seed layer solution was applied to the cleaned silicon substrates. A two-step spin coating was used for a uniform film coating. To achieve a homogenous ZnO seed layer, successive applications of 1000 rpm for 10 s and 1500 rpm for 40 s were used. After deposition, the substrates were heated at 120 °C for 20 min, and the procedure was repeated twice. Later, the substrates coated with the zinc oxide seed layer were placed in a tungsten boat and kept inside a furnace tube at a high temperature (500 °C) for annealing for 2 h. Furthermore, by fabricating multiple samples of ZnO NRs using the same process and comparing the diameters of the as-grown ZnO NRs on the Si substrates, the repeatability of the ZnO seed layer was confirmed. The uniformity of the diameter distribution across multiple samples was maintained. Through this method, seed layer thicknesses of around 23.41 ± 0.23 nm and diameters of the NRs of around 128.50 ± 0.66 were maintained constantly in all our samples. In the supporting information file, Section S1, the SEM top view and cross-section view of the deposited ZnO seed layer, distribution of the seed layer thickness, and distribution of NR diameters are provided.

### 2.2. Preparation of ZnO and Ag-Decorated ZnO NRs

**Figure 1:**
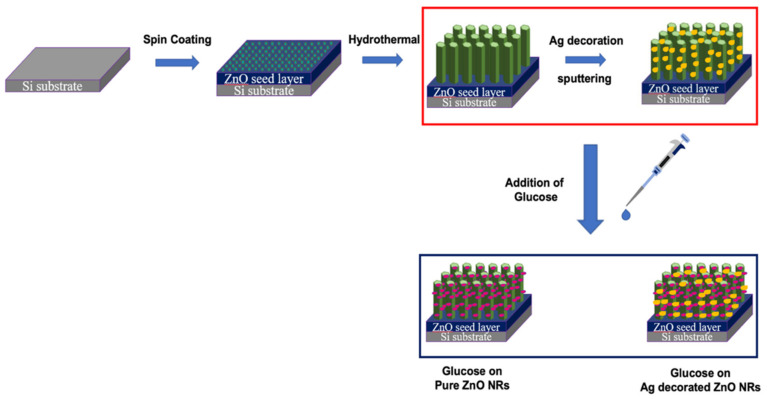
Schematic diagram of the synthesis of ZnO NRs and Ag-decorated ZnO NRs, and the fabrication of electrodes for CV measurement with glucose on ZnO/Ag-decorated ZnO NRs grown on Si substrates.

A stoichiometric ratio of zinc acetate dihydrate and cyclo-hexamethylenetetramine (HMTA) were taken in 90 mL of deionized water and stirred well for 30 min to complete the synthesis of the reaction solution of ZnO NRs. Then, the solution was transferred to the Teflon-lined autoclave and the substrates were kept inside the solution for the growth of the ZnO NRs. The autoclave was placed in an oven at a temperature of 80 °C for 4 h, and after being reduced to room temperature, the substrates were taken out and cleaned with deionized water. The decoration of the ZnO NRs with Ag nanoparticles was achieved using a simple vacuum sputtering technique under ambient conditions for 5 min, adopting the method followed by Yoon, J. et al. [26]. The pressure during the sputtering process was maintained at 20 Pa and the current was controlled at 2 mA. The calculated average diameter of the Ag NPs of 15.55 ± 3.71 nm was maintained using the above-mentioned sputtering conditions for all the samples fabricated in this study. The diameter distribution of the Ag NPs decorating the ZnO NRs is included in the supporting information file, Section S2. After Ag decoration, 20 μL of the glucose solutions of different concentrations was filled with ZnO NRs and placed on a heating plate at 80 °C for 1 h and then used for the analysis. The schematic diagram of the synthesis of ZnO, Ag@ZnO, and glucose/Ag@ZnO is shown in Figure 1.

### 2.3. Characterization Instruments

The crystalline nature and phase structures were investigated using HR-XRD (X’Pert Pro MRD, PANanalytical). The morphology and structural and elemental analysis were evaluated with the FE-TEM system (JEOL JEM-2010) and FE-SEM (ZEISS ULTRA PLUS), which is equipped with an energy dispersive spectrometer (EDS). The structural composition and oxidation state analyses were undertaken with XPS (PHI Quantera II). The Raman vibrational spectroscopy analysis was undertaken with the 3D Nano-Raman Fluorescence Microscope system (3D Raman), Tokyo Instruments Inc., with a 488 nm wavelength semiconductor layer system. The electrochemical performance measurements were carried out on a three-electrode configuration in an aqueous electrolyte of 1 M KOH, tested in the Metrohm Autolab (PGSTAT302 N) multichannel potentiostat/galvanostat. The electrode cell system consists of a working electrode (M41/m-Co_3_O_4_), a (Pt wire) counter electrode, and a (saturated calomel electrode) reference electrode. The fabricated ZnO/Ag@ZnO NRs were used as the working electrode.

## 3. Results and Discussion

**Figure 2:**
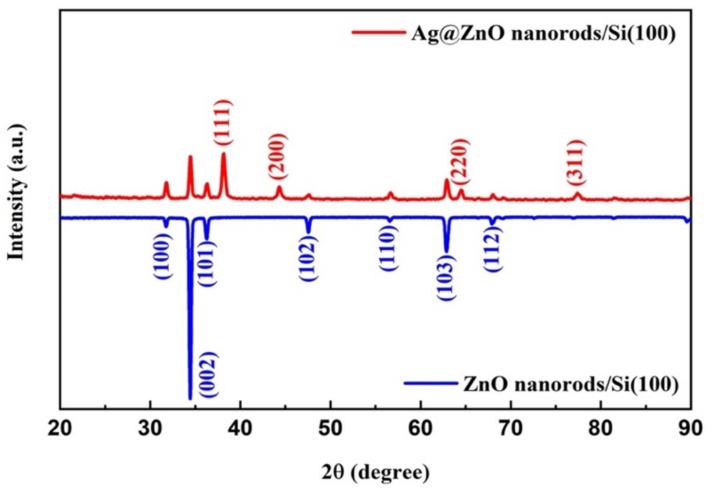
XRD patterns of ZnO and Ag@ZnO NRs grown on Si substrates.

The structural properties of the prepared ZnO and Ag@ZnO NRs were analyzed using an X-ray diffractometer (XRD). The recorded pattern is depicted in Figure 2. The recorded XRD patterns exhibit all the standard diffraction planes for pure and Ag-decorated ZnO NRs, and several crystalline Ag diffraction peaks are overlapped with the ZnO diffraction peaks. The prepared ZnO NRs belong to the hexagonal phase of the standard wurtzite structure, which perfectly matches the JCPDS File Card No. 36-1451 [27,28]. The obtained patterns did not exhibit any other reflection related to any impurity or any form of phase change after the Ag decoration of the as-grown ZNO NRs. Moreover, the highly crystalline phases of the ZnO and Ag@ZnO NRs were further supported by sharp diffraction peaks [29,30].

**Figure 3:**
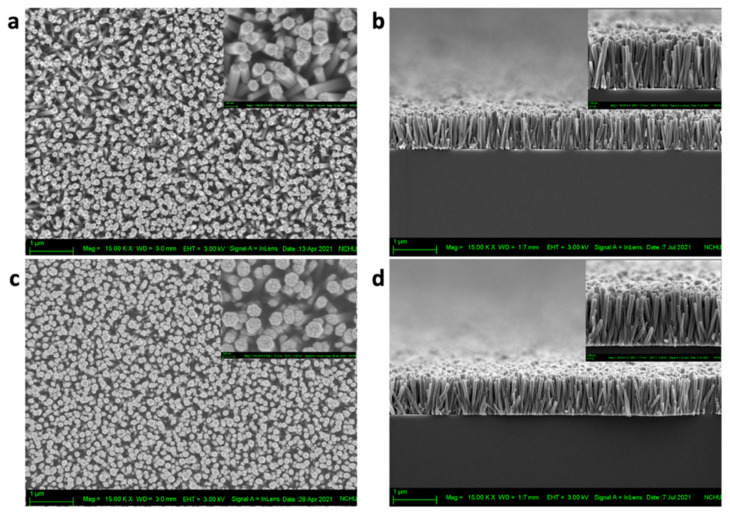
Top and cross-sectional images of (**a**,**b**) ZnO NRs and (**c**,**d**) Ag@ZnO NRs.

The surface morphology, dimensionality, and density of the ZnO NRs were investigated using a field-emission scanning electron microscope (FESEM) and are shown in Figure 3. Figure 3a–d show the top view and cross-sectional view of the ZnO and Ag@ZnO NRs grown on Si substrates with a uniform coverage density, smoothness of the top surface, uniform height, and vertical alignment along the C-axis, which also support and validate the results obtained by XRD. The surface energy of the “activated centers” is higher than that of the nonpolar planes and energetically favors Ag deposition. Furthermore, it can be attributed to the single-ionized oxygen vacancies (V^o+^) on the top or the side of the ZnO NRs [31].

**Figure 4:**
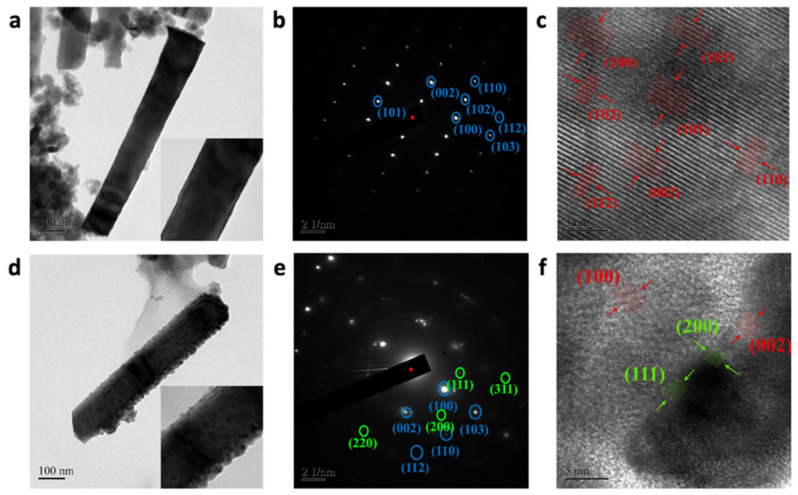
TEM image of a single NR, and SAED pattern and HR-TEM image of a lattice plane of (**a**–**c**) pure ZnO and (**d**–**f**) Ag@ZnO NRs.

Figure 4a–f, the TEM photographs, show the single ZnO and ZnO NRs sputtered with Ag nanoparticles, along with the selected area diffraction pattern (SAED) and high-resolution lattice structure. After the decoration of the ZnO NRs with silver nanoparticles, the formed Ag-ZnO NRs effectively retained their morphology, and the silver nanoparticles were attached to the surface of the ZnO NRs (inset: Figure 4d). A mixture of cubic-phase silver (JCPDS card no. 04-0783) and hexagonal-phase ZnO were observed in the Ag@ZnO NRs from the SAED analysis, while the XRD of both the pure and Ag@ZnO NRs only showed the single hexagonal phase. This is attributed to the interface formation of Ag and ZnO NRs, which was limited in the XRD measurement, which only provides the average information derived from the large area scan.

**Figure 5:**
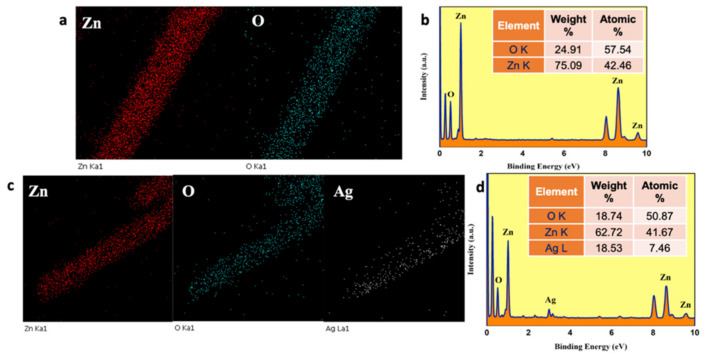
TEM-EDS mapping and spectra with the elemental composition percentage of (**a**,**b**) pure ZnO and (**c**,**d**) Ag@ZnO NRs.

Figure 4b,e, the SAED patterns, show discrete diffraction spots, which indicate the single crystalline nature of the prepared NRs. The marked places that depict the ZnO NR lattice planes are (101), (002), (102), (110), (100), (112), and (103). Meanwhile, the Ag-ZnO NR lattice planes are (110), (103), (112), and (002). These lattice planes coincide with the preferred direction of growth. The compositions of the ZnO and Ag-ZnO NRs were analyzed using energy-dispersive X-ray spectroscopy (EDX). The EDX mapping shows the presence of Zn, O, and Ag elements, and EDX spectra were also recorded for the same region, which confirmed the presence of each element by the appearance of their corresponding peaks (Figure 5a,d). Hence, the results further confirm that the materials were formed without any impurities.

**Figure 6:**
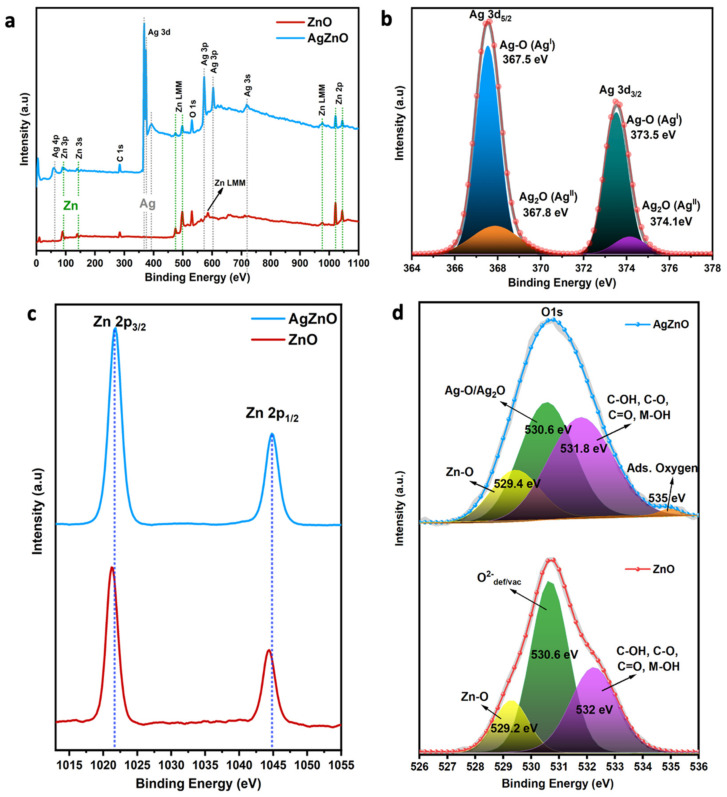
XPS spectra of the (**a**) survey scan, (**b**) Ag 3d core scan, (**c**) Zn 2p core scan, and (**d**) O 1s core scan of pure ZnO and Ag@ZnO NRs.

To evaluate the surface components and chemical states of the pure and Ag@ZnO samples, XPS analysis was also carried out, and the results are shown in Figure 6a–f. The survey scan depicts the presence of Zn, O, and Ag, and no impurity peaks of any other elements were observed. The formation of ZnO NRs was confirmed by the Zn on the surface, present in the form Zn^2+^ in the ZnO structure. The Zn 2p_3/2_ and Zn 2p_1/2_ peaks are located at 1021.70 eV and 1044.80 eV, respectively (Figure 6c). The shift in the Zn 2p peaks towards a higher binding energy in AgZnO is due to the difference in the electronegativity (χ) of Zn (χ = 1.65) and Ag (χ = 1.93). The higher electronegativity of Ag suppressed the valance electron density of Zn in the Zn-O-Ag/Ag-O-Zn bond compared to the Zn-O-Zn bond. This, in turn, weakened the screening effect of Zn and, consequently, a shift in Zn 2p binding energy was observed in the AgZnO sample [32,33].

As shown in Figure 6b, the binding energies of Ag 3d_5/2_ and Ag 3d_3/2_ were observed in the Ag@ZnO sample. The Ag 3d_3/2_ peak is located at ~373.58 eV, and that of Ag 3d_5/2_ is positioned at 367.57 eV. These were attributed to Ag^0^ and Ag^+^. The binding energy of the Ag 3d spectrum in the AgZnO sample was slightly shifted compared with the Ag metal binding energy (Ag 3d_5/2_: 368.2 eV and Ag 3d_3/2_: 374.2 eV). This shift is attributed to the electron transfer from Ag to ZnO through bonding with oxygen (AgO/Ag_2_O). It was also found that the Ag atoms were in mixed oxidation states with two nonequivalent sites, forming bonds with two and four neighboring oxygen atoms [34,35]. Similarly, Gaarenstroom et al. observed the lower binding energy shift in several Ag compounds (AgO/Ag_2_O, AgI, and AgF) compared with Ag metal, and this was attributed to the large extra-atomic relaxation energy effect induced by the electronegative atoms bonding with Ag [34]. The carbon 1s core scan spectra of pure and Ag-decorated ZnO NRs are shown in the supporting information file in Section S3, along with the deconvolution of the peaks which visualize the contributions of C-C, C-H, and C=O bonding features present in the synthesized samples. Figure 6d displays the oxygen 1s core scan spectra of the pure and Ag@ZnO nanostructures. After deconvolution, four types of oxygen binding state were identified in the O 1s core scan. The peak centered at ~529 eV corresponds to the formation of a Zn-O bond with an O^2−^ oxidation state. The AgO and Ag_2_O bonding state is attributed to the peak at 530.6 eV, which originates from the oxygen deficiency sites in the pure ZnO NR sample. The peak positioned at ~532 eV is the response from the multiple combinations of carbon–oxygen bonds in organic compounds. Additionally, a weak chemisorbed surface oxygen bonding state was observed at ~535 eV, which only appeared in the Ag@ZnO NR sample [36,37,38].

**Figure 7:**
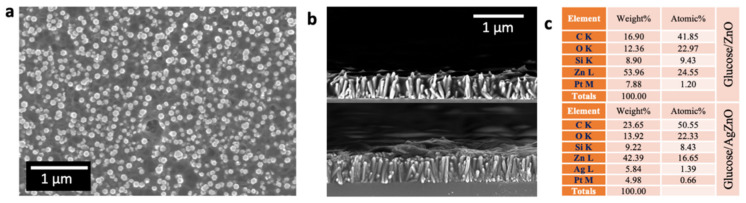
SEM image and EDS spectra with elemental composition table. (**a**) Top view of glucose/pure ZnO NRs, (**b**) cross-sectional view of glucose/pure ZnO NRs (top) and glucose/Ag-decorated ZnO NRs (bottom), and (**c**) EDS elemental composition percentage table.

**Figure 8:**
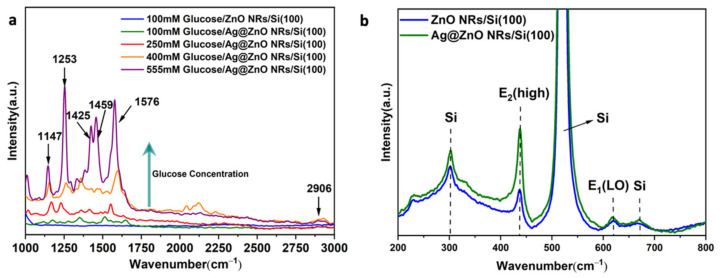
(**a**) Glucose concentration dependence Raman spectra of pure and Ag-decorated ZnO NRs. (**b**) Raman spectra of pure ZnO and Ag@ZnO NRs visualizing the inorganic molecule vibrations in the wavelength range between 200 and 800 cm^−1^.

As the preliminary characterization results validated the quality of the synthesized samples, the main objective, the glucose sensing property, was examined by drop casting the glucose solution with various concentrations. Figure 7a,b depict the top and cross-sectional SEM views of the glucose-coated ZnO and Ag-decorated ZnO NRs on silicon substrates. The EDS atomic composition percentage (Figure 7c) confirms the presence of glucose compound that is uniformly spread over the NRs. Figure 8a,b show the Raman spectra of the pure and Ag@ZnO NRs. The peaks at 97, 226, 301, 438, 520, 618, and 669 cm^−1^ are attributed to the E2 (low), 2E2 (low), 2E2 (low)- 2E2 (low), E2 (high), 2B1 (low), A1 (LO), and TA + LO modes, respectively, and correspond to the ZnO and Ag@ZnO NRs. These vibrations confirm the wurtzite structure of ZnO. The 1000–3000 cm^−1^ vibration region, which belongs to the organic molecules’ modes, showed an enhanced Raman signal after the decoration of the Ag NPs. However, the region from 200 to 700 cm^−1^ was deconvoluted to the six identified vibration modes, of which two belong to Ag nanoparticles. The first one, localized at 226 cm^−1^, corresponds to Ag-O_2_ bonds formed by a chemisorption process due to surface defects in the metallic silver. The second one, at 438 cm^−1^, is directly attributed to the interfacial surface phonon mode originating from Ag deposition on the ZnO surface. Qualitatively, the Raman spectra alone provided information on the average presence of glucose, according to their concentrations, due to the enhancement of the electrochemical activity by surface plasmon resonance originating from the Ag decoration [39]. Furthermore, the UV transmission and absorption spectra and photoluminescence spectra were recorded for the as-grown pure ZnO and Ag-decorated ZnO NRs to realize the optical electron transitions between the bands, and these are included in the supporting information file in Section S4.

**Figure 9:**
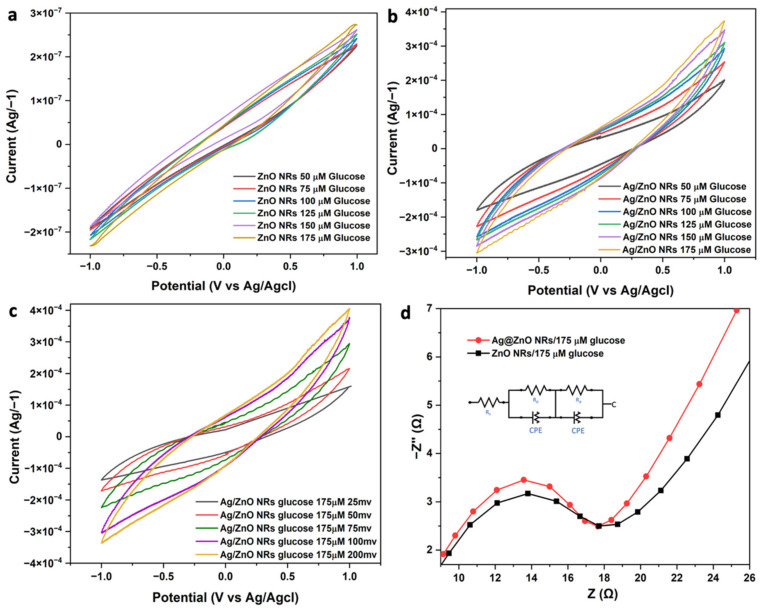
Cyclic voltammograms of (**a**) ZnO and (**b**) Ag@ZnO NR glucose sensor devices at various concentrations of glucose with a constant potential of 100 mV. (**c**) Cyclic voltammograms of Ag@ZnO NR glucose sensor devices at a constant concentration of glucose with various electrode potentials. (**d**) Nyquist semicircle graph recorded using the fabricated sensor devices.

The recorded cyclic voltammograms of the fabricated samples are depicted in Figure 9a–c. EIS measurements were used to monitor the electrocatalytic behavior of each built step in a series of 0.1 M KOH electrolytes, and the impedance data were then calculated using a Randles equivalent circuit (see the inset of Figure 9d).

The ZnO/Ag@ZnO NRs formed on the electrode caused the Nyquist semicircle to shrink and the Ret value to drop (Ret = 101), indicating an increased rate of electron transmission. The Ag-decorated electrode resulted in significantly increased electron transfer rates, such as Ret = 6.98, 13.7, 409, and 10.9 for the Ag-ZnO NR 100 mM glucose, 250 mM glucose, 400 mM glucose, and 555 mM glucose electrodes, respectively. The electrode performance was optimized by dipping a 100 mM glucose-coated Ag-ZnO NR electrode into the precursor solution for 20 s. This increased the electrode’s electron transfer rate (Ret = 6.98).

The data in Figure 9b also show that the CV response of the Ag-ZnO NR electrode was evaluated in a 0.1 M KOH solution on the CVs in the presence of glucose on a different electrode (scan rates, 100 mVs^−1^). It has been determined that the Ag@ZnO sample had a higher anodic peak current density than pure ZnO NRs. The fact that glucose addition to the electrolyte caused a change in the oxidation peak current density for distinct samples stands out as particularly important.

**Figure 10:**
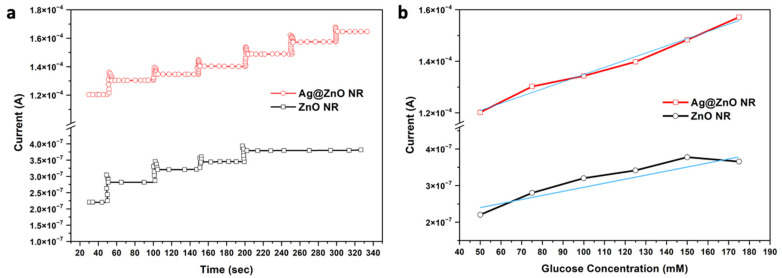
(**a**) Amperometric responses of ZnO and Ag@ZnO NR glucose sensor devices at room temperature with constant potential. (**b**) Linear behavior of sensitivity based on the current response upon addition of glucose at various concentrations for both the ZnO and Ag@ZnO NR sensor devices.

After glucose was added, the peak current of the CV positive scan increased, suggesting that glucose oxidation was enhanced on the electrode. Glucose oxidation on the working electrode surface typically occurs in two phases. In order to generate an oxidative current, glucose molecules must first be oxidized to become gluconolactone, which they accomplish by releasing electrons to the NRs. After that, the gluconolactone is broken down to become gluconic acid. We have successfully proven how to manufacture an excellent non-enzymatic glucose sensor electrode by directly growing ZnO NRs on Si substrates and then altering them with Ag. Ag-ZnO NR electrodes are unique because the ZnO NRs generated directly on the electrode surface have a structure that is easily penetrated and a large surface area for Ag modification, both of which increase the electrochemical activity for glucose detection. In order to demonstrate the analytical parameters (i.e., the sensitivity, linear range, detection limit, and response time), the amperometric responses of the ZnO NR and Ag@ZnO NR electrodes were measured at a fixed voltage of +0.8 V in a 0.1 M KOH solution through the stepwise addition of glucose at different concentrations. Figure 10a shows the amperometric responses of both the ZnO NR and Ag@ZnO NR devices.

The linear sensitivity behavior with respect to the glucose concentration of the fabricated ZnO NR and Ag@ZnO NR electrochemical sensors is displayed in Figure 10b. The measurement of the response current started with a glucose concentration of 50 µM and increased in steps of 25 µM up to 175 µM. The sensitivities of both the bare ZnO NR and Ag-decorated ZnO NR sensors were calculated by determining the slope of the linear line drawn for the measured response current. In the case of the pure ZnO NR device, the response of the sensor to the changes in the glucose concentration are represented by a straight increase in the output current until it reaches the saturation point at 150 µM.

**Figure 11:**
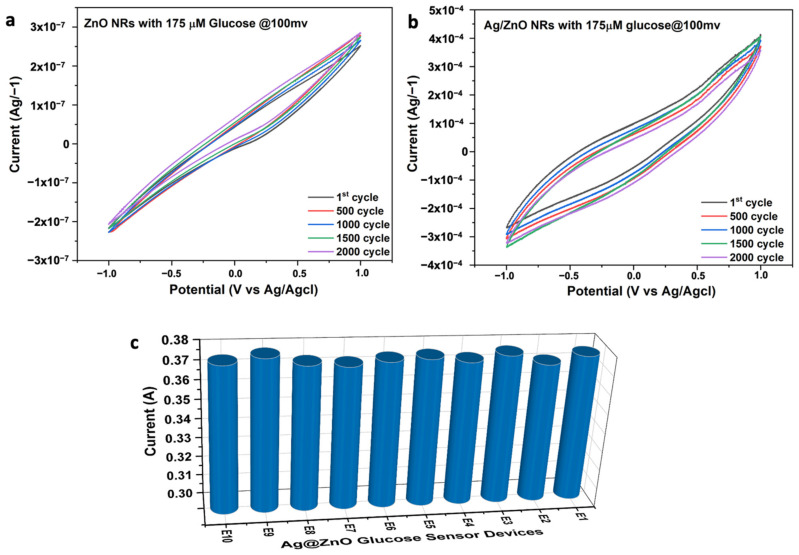
Stability and endurance cycle test results of the fabricated glucose sensor devices: (**a**) ZnO NRs and (**b**) Ag@ZnO NRs. (**c**) Reproducibility test results of different Ag@ZNO glucose sensor devices (E1-E10) showing the consistent current response upon exposure to the constant concentration of glucose.

In the meantime, the Ag decoration increased the saturation level of the sensor. The lower limit of detection, which is the lowest concentration of glucose that can be detected by the device, can be calculated using the same data shown in Figure 10b. The formula used to calculate the lower limit of detection is 3×σ/slope, where σ is the standard deviation of the current density, and it was found to be 1.29 µM and 1.07 mM for the pure ZnO NRs and Ag@ZnO NRs, respectively. The calculated sensor characteristic parameters are listed in Table 1 along with data reported in the literature. It can be observed that the sensor is sensitive to lower concentrations of glucose. Reproducibility, reusability, and stability are other vital parameters for measuring the efficiency of sensing devices. The reusability of the Ag@ZnO electrode was measured with 175 mM of glucose at a 100 mVs^−1^ scan rate, and the obtained data are shown in Figure 11a,b. After 2000 cycles, the Ag@ZnO NR glucose-sensing electrode retained around 99% of its original response, suggesting the excellent reproducibility and reusability of our sensing electrode. Similarly, the reproducibility of the Ag@ZnO NR electrode was investigated by employing 10 freshly prepared non-enzymatic glucose sensors. Their CV responses were recorded in a 0.1 M KOH solution containing 175 mM glucose at a scan rate of 100 mVs^−1^. The peak current of the CV response is presented in Figure 11c. The excellent stability of the electrode was due to the nanorods grown directly on the electrode surface, which provided robust mechanical stability to the sensing device.

## 4. Conclusions

The present study proposed the use of ZnO NRs and Ag-decorated ZnO NRs grown on Si substrates (electrodes) as efficient, reliable, and cost-effective non-enzymatic glucose biosensors. Our study started with the synthesis of ZnO NRs on Si substrates using the simple low-temperature hydrothermal process. FE-SEM was employed to identify the morphology of the as-grown ZnO NRs and the ZnO NRs sputtered with silver nanoparticles. The local cubic sphalerite and hexagonal wurtzite structures in the pure ZnO and Ag-decorated ZnO NRs were revealed by the TEM analysis. The elemental composition and the atomic composition percentage of each element were obtained from the TEM-EDS and EDS mapping analysis. In addition, the elements were identified by XPS, and the sub-peak changes in the peak fitting were observed after sputtering with the silver nanoparticles. The excellent performance of the non-enzymatic glucose sensor electrode was demonstrated. We successfully fabricated the pure ZnO NRs and Ag-decorated ZnO NRs on the Si substrates/electrode, and then the target glucose detection was investigated. The unique sensor design, in which the target glucose was deposited on the nanostructured ZnO/Ag@ZnO electrode with a large surface area, enhanced the electrochemical activity for glucose detection. The highest sensitivity of 2792 µA/(mM·cm^2^) with the lowest detection limit of 1.29 µM was attained with the Ag@ZnO NRs, which were better than the pure ZnO NR devices. We anticipate that the findings of this work will be a useful addition to the literature on non-enzymatic glucose sensors.

## Figures and Tables

**Table 1:** Comparison of the characteristic parameters of the fabricated glucose sensor devices with the published literature.

Nanostructures	Sensitivity µA/(mM·cm^2^)	Detection Limit (µM)	References
ZnO NRs	1.19	1.29	This work
Ag@ZnO NRs	2792	1.07	This work
Ag doped ZnO NRs (Enzymatic sensor)	3.85	1.5	[1]
Ni-ZnO NRs	61.78	2.5	[40]
Co-ZnO nanoclusters	13.3	20	[41]
Al-ZnO film	5.5	160	[42]
Au-ZnO NRs/FTO electrode	4416	0.12	[43]

## Data Availability

The data presented in this study are available on request from the corresponding author.

## Supplementary Materials

The following are available online at https://www.mdpi.com/article/10.3390/nano13040754/s1. Section S1: ZnO Seed Layer Characterization.; Section S2: Ag Nanoparticles Diameter Distribution Graph on ZnO NRs.; Section S3: XPS C 1s Core Scan.; Section S4: Optical Spectroscopy.
