# Supplementary material for: Ag-Decorated Vertically Aligned ZnO Nanorods for Non-Enzymatic Glucose Sensor Applications
Source: Nanomaterials (Basel). 2023 Feb 17;13(4):754. doi: 10.3390/nano13040754 (PMC9965292; doi:10.3390/nano13040754)
Supplement: Supplementary file 1 [file nanomaterials-13-00754-s001.zip › nanomaterials-2204885-supplementary.pdf]

nanomaterials

Supporting Information

Ag-Decorated Vertically Aligned ZnO Nanorods for Non-Enzymatic Glucose Sensor Applications

Yu-Hsuan Lin [1,†], Chandrasekar Sivakumar [2,3,†], Babu Balraj [2,4], Gowtham Murugesan [5], Senthil Kumar Nagarajan [5] and Mon-Shu Ho [1,2,3,*]

[1] Institute of Nanoscience, National Chung Hsing University, Taichung City 40227, Taiwan
[2] Department of Physics, National Chung Hsing University, Taichung City 40227, Taiwan
[3] Innovation and Development Center of Sustainable Agriculture (IDCSA), National Chung Hsing University, Taichung 40227, Taiwan
[4] Department of Physics, KPR Institute of Engineering and Technology, Coimbatore 641407, Tamilnadu, India
[5] Postgraduate and Research Department of Physics, Nanotechnology Lab, Kongunadu Arts and Science College, Coimbatore 641029, Tamilnadu, India
* Correspondence: msho@dragon.nchu.edu.tw; Tel.: +886-4-22840427
† The authors contributed equally to this work.

S1. ZnO Seed Layer Characterization

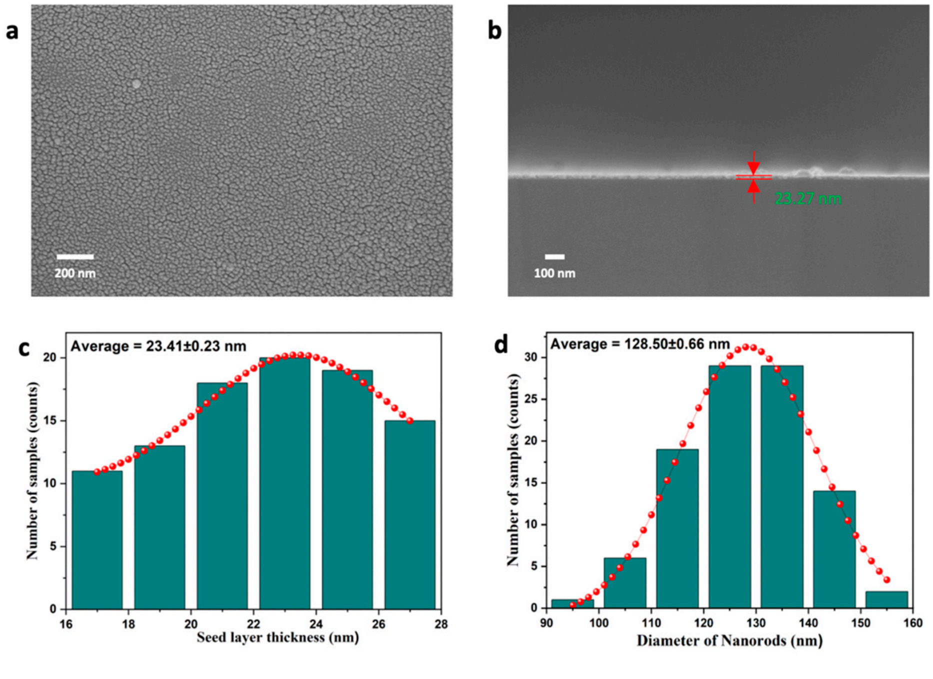

Figure S1. SEM image of ZnO seed layer after annealing at 500 °C **(a)** top view and **(b)** cross sectional view with seed layer thickness measurement. **(c)** Distribution seed layer thickness measured on different samples and at different locations. After the Gaussian fit the average thickness of the seed layer is calculated as 23.41 ± 0.23 nm. **(d)** Distribution of ZnO NRs diameter with gaussian fitting and calculated average diameter is 128.50 ± 0.66.

S2. Ag Nanoparticles Diameter Distribution Graph on ZnO NRs

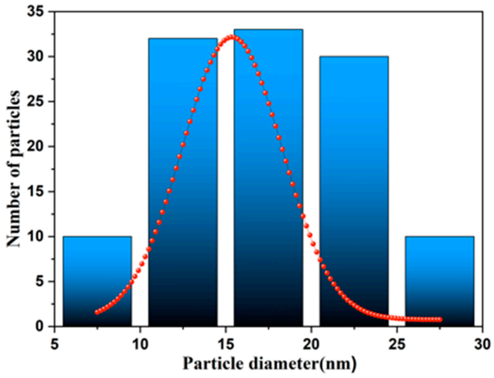

Figure S2. Ag NPs diameter distribution decorated on the ZnO NRs with the average diameter of 15.55 ± 3.71 nm.

S3. XPS C 1s Core Scan

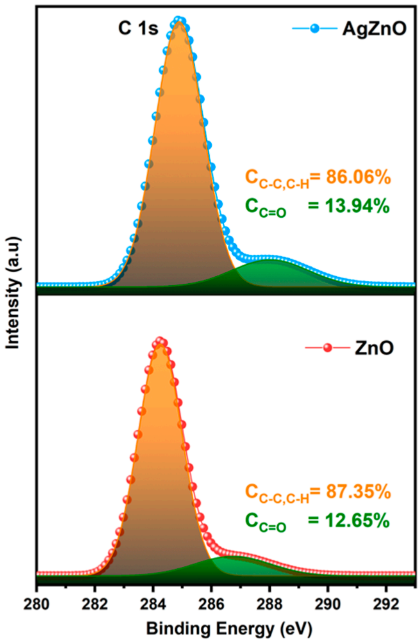

Figure S3. C 1s core scan spectrum of ZnO and Ag@ZnO NRs.

S4. Optical Spectroscopy

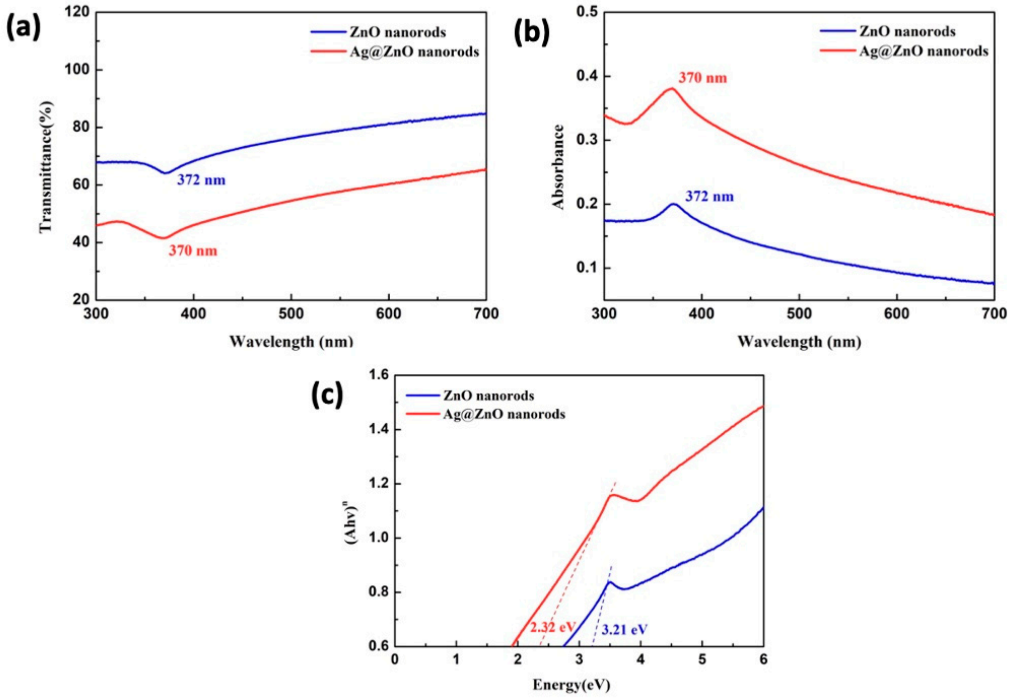

Figure S4. (a) UV transmission **(b)** Absorption spectra and **(c)** Optical energy gap diagram of ZnO and Ag@ZnO NRs.

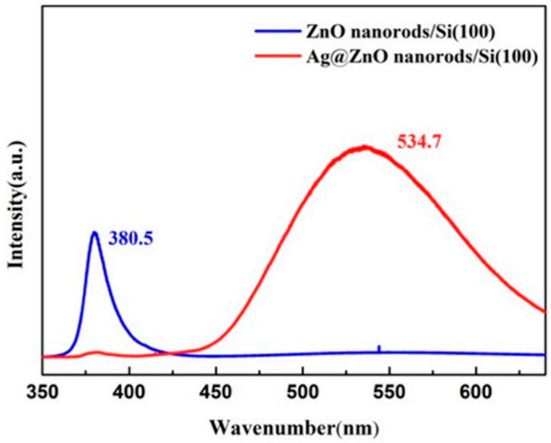

Figure S5. PL fluorescence spectra of ZnO and Ag@ZnO NRs.
